# Recovery of uranium from phosphate ore in the Sheikh Habil-Iran mine: part I— multivariable optimization of leaching process using the response surface method

**DOI:** 10.3389/fchem.2023.1292620

**Published:** 2023-12-06

**Authors:** A. Abdeshahi, M. Outokesh, D. Ghoddocy Nejad, M. Habibi Zare, M. H. Sadeghi

**Affiliations:** ^1^ Department of Energy Engineering, Sharif University of Technology, Tehran, Iran; ^2^ Nuclear Fuel Research School, Nuclear Science and Technology Research Institute, AEOI, Tehran, Iran; ^3^ Jaber Ebne Hayyan National Research Laboratory, NSTRI, Tehran, Iran

**Keywords:** uranium recovery, phosphate ore, optimization, leaching process, Sheikh Habil-Iran mine, flotation, calcination of phosphate ores

## Abstract

In this research, the recovery of uranium from the phosphate ore of the Sheikh Habil-Iran mine using flotation/calcination-leaching processes has been investigated. A 75–150 μm phosphate ore particle size, sodium oleate as a collector with a concentration of 2,000 g/ton of rock, pH = 10, and 5 min flotation time were obtained as the optimum parameters of flotation using the reverse method, leading to phosphate ore with a grade of 180 ppm UO_2_, 36.1% P₂O₅, 7.22% SiO_2_, and CaO/P₂O₅ = 1.23. The optimum calcination parameters were selected as 100 μm phosphate ore particles size at D80, 900°C temperature, and 2 h heating time, which resulted in phosphate ore with a grade of 173 ppm UO_2_ and 31.9% P₂O₅. An L/S (liquid to solid ratio) = 5, 3 M sulfuric acid concentration, 80°C temperature, and 5 h leaching time were obtained as the optimum leaching parameters using the response surface methodological approach. The efficiency of uranium recovery from phosphate ore pre-treated by flotation and calcination methods was 84.2% and 75.2%, respectively. The results indicated that flotation has superiority over calcination as a pre-treatment method of phosphate ore in the Sheikh Habil-Iran mine.

## Highlights

1) Recovery of uranium from phosphate ore of Sheikh Habil-Iran mine
2) Implementation of RSM approach for optimization of uranium extraction
3) Leaching process select for uranium recovery from phosphate ore
4) Complex experiments were performed with four parameters (L/S, Concentration, Time and Temperature) of effective

## 1 Introduction

Today, uranium is mainly obtained from primary resources (Fatemi et al., 2019; Fatemi et al., 2020a; Fatemi et al., 2020b; Sadeghi et al., 2020; Sadeghi et al., 2021). Due to the depletion of primary resources, the world has turned to secondary resources such as phosphate ores, sea water, petroleum coke, etc. Approximately 9,700 tpa uranium oxide can be extracted from phosphoric acid (Beltrami et al., 2013; Kawatra and Carlson, 2013; Beltrami et al., 2014; Arhouni et al., 2023; Lammers et al., 2023).

The amount of phosphate reserves in the world is estimated to be 71 billion tons. Phosphate ores are mainly divided into sedimentary and igneous, where the amount of sedimentary phosphates is close to 95% (Pufahl and Groat, 2017). Phosphorite deposits are chemically formed in a relatively deep marine environment under calm conditions. The phosphorus and uranium content in marine phosphorite horizons, such as those found in a formation like the Pabdeh Formation, do not show significant variations over distances of several kilometers. Sedimentary phosphorite deposits contain uranium-bearing fluorapatite (francolite) concretions, where uranium replaces calcium during the formation stages, forming uranium-containing complexes. Therefore, only sedimentary phosphorites, based on their sedimentary nature and origin, contain uranium. Sedimentary phosphate ores with a low phosphate grade (less than 30%) require some pre-treatment to remove tailings before producing phosphoric acid. The type of pre-treatment depends on ore properties such as density, particle size, morphology, surface chemistry, magnetic properties, electrical conductivity, color, and porosity of particles (Ptacek, 2016). Today, most (more than 75%) phosphate ores are processed using the wet-phosphoric acid (WPA) process, with sulfuric acid as the digestive acid. Prior to this process, the phosphate ore needs to be beneficiated or pre-concentrated, which is mostly performed using flotation or calcination (Kawatra and Carlson, 2013; Zhang et al., 2023).

**FIGURE 1:**
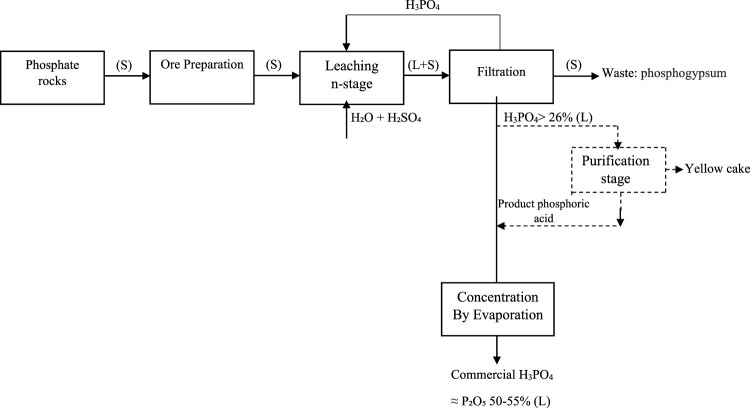
Schematic of phosphoric acid production from phosphate ore.

A schematic of phosphoric acid production from phosphate ore is shown in Figure 1 (Beltrami et al., 2014; Haneklaus et al., 2017; Steiner et al., 2020). After ore preparation, the ore is leached to produce a phosphoric acid solution. As mentioned above, approximately 75% of the global phosphoric acid industries use sulfuric acid for leaching phosphate rocks, as shown in reaction 1, where the sulfuric acid attacks the carbonate minerals to precipitate calcium sulfate and produce phosphoric acid (Beltrami et al., 2013; Beltrami et al., 2014).

$${Ca}_{5}F{\left(PO_{4}\right)}_{3}\left(s\right)+5H_{2}{SO}_{4}\left(aq\right)+10H_{2}O\left(aq\right)\to 3H_{3}{PO}_{4}\left(aq\right)+5CaSO_{4}.2H_{2}O\left(s\right)+HF\left(aq\right)$$ (1)

Another method for producing phosphoric acid is the dry method. To produce food-grade phosphoric acid, the phosphate ore is first reduced with coke in an electric arc furnace to give elemental phosphorus. Silica is also added, resulting in the production of calcium silicate slag. Elemental phosphorus is distilled out of the furnace and burned with air to produce high-purity phosphorus pentoxide, which is dissolved in water to give phosphoric acid.

Finally, phosphoric acid produced with an approximate concentration of 24%–28% P_2_O_5_ is considered a feed for the recovery of uranium from WPA using various methods such as solvent-extraction, ion-exchange, and membrane separation (Bautista, 1993; Kabay et al., 1998; Beltrami et al., 2014).

**FIGURE 2:**
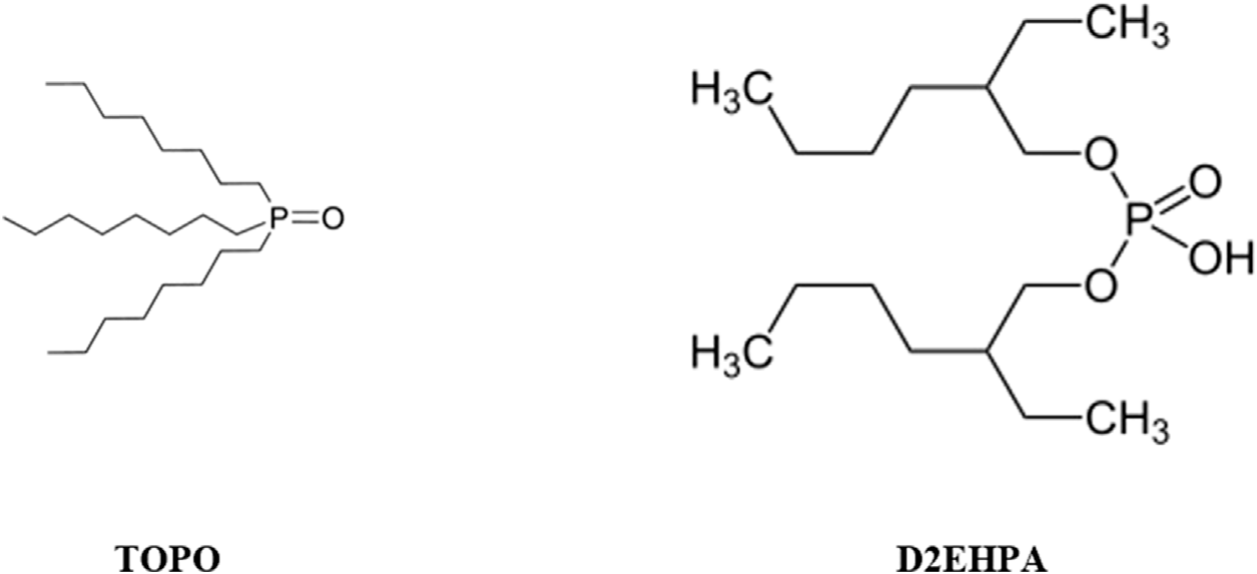
Molecular schematics of D2EHPA and TOPO solvents.

One of the most famous and widely used methods is solvent extraction (SX), which is an efficient process for the recovery of hexavalent uranium from a phosphoric acid medium (Yan-Zhao et al., 2002; Beltrami et al., 2013). In this regard, different organic solvents have been tested to date, among which the synergistic mixture of di-2-ethylhexyl phosphoric acid (D2HEPA) and tri-octyl-phosphine oxide (TOPO) are the typical solvents used to separate uranium, see reaction 2 (Ali et al., 2012; Beltrami et al., 2012; Beltrami et al., 2013; Beltrami et al., 2014). The molecular formulas of D2EHPA and TOPO are displayed in Figure 2.

$$UO_{2}^{2+}+2{\left(HL\right)}_{2}^{-}+TOPO\to UO_{2}^{-}{\left(HL\right)}_{2}L_{2}^{-}TOPO+2H^{+}$$ (2)

where HL and L are the monomeric and deprotonated forms of D2EHPA, respectively.

The extraction of other metallic elements using solvent extraction complexes, such as D2EHPA + TOPO, depends on specific conditions such as the concentration of metallic elements and operational parameters. These elements are typically considered side impurities, with iron (Fe), nickel (Ni), aluminum (Al), and cobalt (Co) the most commonly known. However, generally, there is a much greater inclination toward extracting uranium (U) in these complexes compared to other metallic elements. This high preference is based on the different physicochemical properties of the metallic elements and the formation of various complexes with D2EHPA + TOPO.

Furthermore, various separation techniques can be employed for the separation of these side metallic elements. These techniques include the use of dual-extraction solvents, ion exchange technologies, adsorption processes, and utilization of electrochemical methods.

In this research, the production of phosphoric acid and the recovery of uranium from the phosphate ore of the Sheikh Habil-Iran mine has been investigated. Flotation and calcination as pre-treatment processes for the phosphate ore are used and optimized separately. The pre-treated phosphate ore was leached using sulfuric acid. The leaching parameters were optimized to produce a suitable grade of phosphoric acid and to maximize the uranium recovery using a response surface methodological approach (RSM). The solvent extraction of uranium from phosphoric acid and the precipitation of ammonium uranyl carbonate are described in the second part of the research, published separately.

## 2 Materials and methods

### 2.1 Geological origin

The Sheikh Habib phosphate deposit is composed of fluorapatite (62%), occurring as well-rounded and moderately sorted phosphate pellets. Phosphate is also found as apatite in echinoid fragments and phosphate-rich nodules. Additionally, a secondary process called cementation has occurred in the deposit, resulting in particle bonding and the formation of cement. The cement includes carbonate cement (calcite and some dolomite) and minor amounts of anhydrite cement.

According to the results of XRD analysis, the major gangue minerals in the deposit include calcite (27%–30%), quartz (6%–9%), and clay minerals such as illite and muscovite (1%).

The geological origin of the Sheikh Habib phosphate deposit can be attributed to various geological factors and processes that have contributed to its formation and evolution.

### 2.2 Materials

Sodium oleate (C_18_H_33_NaO_2_), sodium sulfate (Na_2_SO_4_), potassium hydroxide (KOH), hydrochloric acid (HCl), soda (NaOH), sodium silicate (Na_2_SiO_3_), citric acid (C_6_H_8_O_7_), methyl isobutyl carbinol (MIBC), and starch (C_6_H_10_O_5_)_n_ were purchased from Sigma-Aldrich**.** Sulfuric acid (98 wt%) was purchased from Kimia Tehran Acid Co. Phosphoric acid (H_3_PO_4_) was obtained from the Razi factory.

### 2.3 Analysis and characterization

X-ray fluorescence spectrometry (XRF, Hitachi SEA 1000A) was used to determine the composition of the phosphate ore. The Hitachi SEA 1000A XRF device is an X-ray fluorescence analyzer used for the analysis of chemical elements and compounds in samples. This device offers high precision, a wide range of analyzable elements, high analysis speed, and user-friendliness. X-ray fluorescence technology can detect various elements and chemical compounds in samples, providing analysis results with ppm levels of accuracy. The Hitachi SEA 1000A device is suitable for applications that require rapid and accurate analysis of different chemical elements and compounds in samples.

The uranium concentration was measured using inductively coupled plasma spectrometry (ICP-OES, PerkinElmer 2000 DV). The PerkinElmer 2000DV is a laboratory instrument used for the analysis of chemical elements in samples. It utilizes ICP-OES technology, combining inductively coupled plasma and optical emission spectroscopy, to identify the elements present in a sample and determine their concentrations. The PerkinElmer 2000DV offers high precision, sensitivity, a broad range of analyzable elements, fast analysis speed, and automation capabilities. It finds applications in various fields, including analytical chemistry, earth sciences, environmental chemistry, food and pharmaceutical industries, agriculture, and mining. The crystalline phase structure of the phosphate ore was characterized by X-ray diffraction (XRD, Philips PW1800), with a Cu-kα radiation source filtered through a nickel sheet. The Philips PW1800 XRD device utilizes X-ray radiation to analyze the crystal structure of samples. By exposing the sample to X-ray radiation, a diffraction pattern is formed, revealing the structural characteristics of the sample. This device offers high precision and resolution, enabling the analysis of complex patterns. Additionally, it can investigate structural changes in response to varying experimental conditions. The Philips PW1800 device finds applications in various fields such as materials science, chemistry, physics, metallurgy, environmental science, and earth sciences.

### 2.4 Apparatus

A rod mill (Taifazarin TA-12000 RAB) and a ball mill (NARYA–PGM 800) were used to crush the ore. A mechanical sieve shaker (MG Scientific Ro-Tap) was used to grind the crushed ore. A sampler (Taifazarin RSD-500) was applied to take 500 g samples of the sieved ore. A laboratory thermal furnace (Nabertherm LT9 12 B410) was used for ore calcination. A 4 L mechanical flotation system (Metso D12 VFD) was used for the flotation process. A three-necked glass flask equipped with a mechanical agitator, a glass reflux condenser, and a thermometer was used as a leaching reactor. The reactor was heated using an electric heater.

### 2.5 Procedures

The mineral ore was initially crushed by a rod mill in an open-air environment at a speed of 350 rpm for 60 min. This process reduces the dimensions of the ore to below 10 mm. Then, the mineral ore was ground using a ball mill at a speed of 150 rpm for 15 min and eventually undergoes sieving processes. This process generates particles with intermediate sizes in the range of 75–150 μm (Gallala et al., 2016) and 100 μm (Abouzeid, 1980; Abouzeid, 2008) to facilitate the flotation and calcination processes. In the sieving process, the mineral particles are separated based on their physical and chemical properties, to be used as the final product or transferred to subsequent stages of mineral processing.

#### 2.5.1 Calcination

The milled sample (100 μm) was calcined in a thermal furnace at 900°C for 1–3 h (Abouzeid, 1980; Abouzeid, 2008). The calcined sample was naturally cooled to ambient temperature. The operating conditions, such as temperature, size, and time for the calcination process are similar to industrial practices; however, the process is conducted in batch mode rather than continuous.

#### 2.5.2 Flotation

At first, 500 g milled sample (75–150 μm) was mixed with 1,500 mL distilled water in the chamber of the flotation machine at a stirring speed of 1,200 rpm (Al-Fariss et al., 1993). Then, the pH of the solution was adjusted using HCl (32 wt%) and NaOH (Clifford et al., 1998). After adjusting the pH, 250 mL depressant was poured into the solution. After 5 min, 250 mL collector was poured into the solution. After another 5 min, an MIBC solution at a rate of 2,000 g. ton^−1^ of rock was added to the solution to make a froth. After 4 min, aeration was performed at a flow rate of 200 cm^3^ min^−1^ for 5 min. The floated part was removed from the top of the column and the residue remained (Al-Fariss et al., 1993). The floated and tail phases were filtered, dried, and weighed. Moreover, the conditions and materials used in the mentioned flotation process are similar to industrial practices. However, the process conducted in the laboratory setting is carried out in batch mode rather than continuous.

#### 2.5.3 Leaching

**FIGURE 3:**
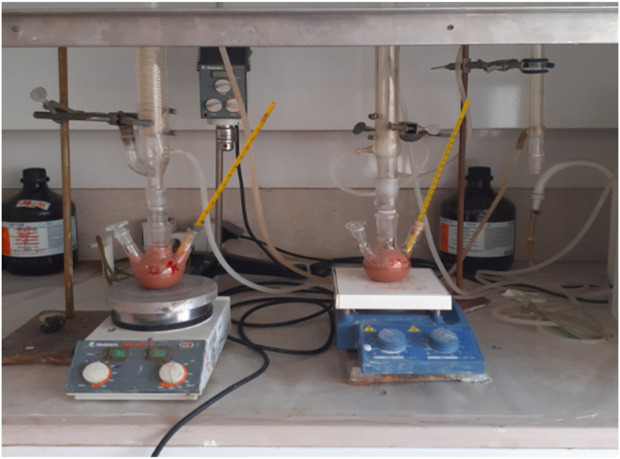
Image of the phosphate ore leaching process in the laboratory and on a batch scale.

The leaching process was carried out in the laboratory as a batch implementation, which is depicted in Figure 3. At first, sulfuric acid (2–6 M) was charged into the reaction vessel. Next, the vessel was heated to approximately 40°C–100°C and the pre-treated ore sample was added to the solution. The liquid-to-solid ratio was set at 2–6 mL/g. The agitation speed of the reactor was 400 rpm.

Several leaching stages were conducted to produce phosphoric acid with a grade of 26% P_2_O_5_. To increase the efficiency of the leaching stages, the intermediate gypsums were washed with 12 wt% sulfuric acid. The washing solution and fresh pre-treated ore were fed into the reactor for the next step.

The leaching efficiency (E) was calculated using Equation (3):

$$E\left(\%\right)=\left(\frac{V_{L}\times C_{L}}{\left(m_{S}\times C_{S}\right)+\left(V_{L1}\times C_{L1}\right)}\right)\times 100$$ (3)

where V_L_ is the leach liquor volume (l), C_L_ is the uranium concentration of the leach liquor (mg/L), m_s_ is the ore mass (kg), C_S_ is the uranium concentration of the ore (g/ton), V_L1_ is the initial solution volume (l), and C_L1_ is the uranium concentration of the initial solution (mg/L).

## 3 Results and discussion

### 3.1 Characterization of the phosphate ore

The chemical compositions of the Sheikh Habil-Iran ore are tabulated in Table 1. The results indicate that the calcium, silicon, iron, aluminum, and manganese oxides are the main impurities in the ore. The fraction of UO_2_ was 159 ppm, which is attractive as a secondary resource of uranium. The XRD analysis of the phosphate ore shown in Table 2 indicates that Ca_5_(PO_4_)_3_F (Fluorapatite), CaCO_3_ (Calcite), and SiO_2_ (Quartz) formed the crystalline phase of the ilmenite concentrate. Ilmenite is a mineral that consists of a mixture of fluorapatite, calcite, and quartz. Fluorapatite is a chemical compound that contains calcium, phosphate, and fluorine. Calcite is a mineral composed of calcium carbonate, while quartz is a mineral made up of silicon dioxide. These three substances are commonly found in ilmenite and are present as small crystals within the structure of ilmenite.

**TABLE 1:** XRF analysis of the chemical composition of the Sheikh Habil-Iran ore.

Components	P_2_O_5_	UO_2_	CaO	SiO_2_	Fe_2_O_3_	Al_2_O_3_	MgO	SO_3_	LOI
wt%	29.49	0.0159	46.24	8.17	3.37	0.94	0.47	0.26	9.89

**TABLE 2:** XRD analysis of the Sheikh Habil-Iran ore.

Name	Chemical formula	(wt%)
Fluorapatite	Ca_5_(PO_4_)_3_F	62
Calcite	CaCO_3_	28
Quartz	SiO_2_	9
Muscovite –Illite	KAl_2_Si_3_AlO_10_(OH)_2_	1

### 3.2 Calcination

**FIGURE 4:**
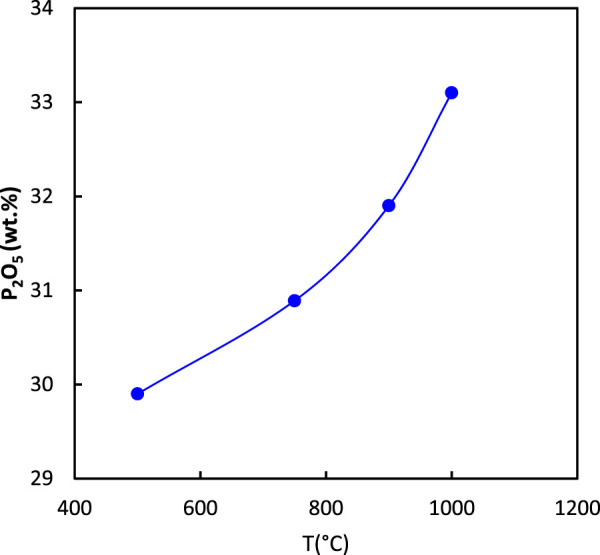
Effect of calcination temperature on the percentage of P_2_O_5_ in the ore (2 h calcination).

**FIGURE 5:**
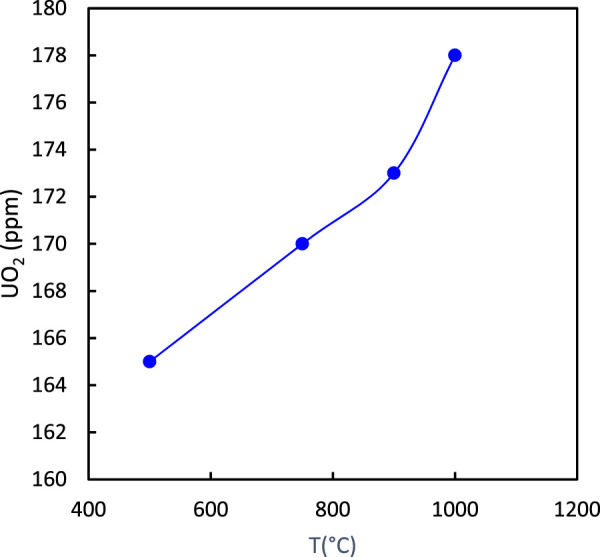
Effect of calcination temperature on the percentage of UO_2_ in the ore (2 h calcination).

The effect of calcination temperature on P_2_O_5_ and UO_2_ is displayed in Figures 4, 5, respectively. The results indicated that an increase in calcination temperature increased the percentage of P_2_O_5_ and UO_2_ in the ore. From energy consumption and environmental standpoints, a temperature of 900°C was considered economical (Abouzeid, 1980).

**FIGURE 6:**
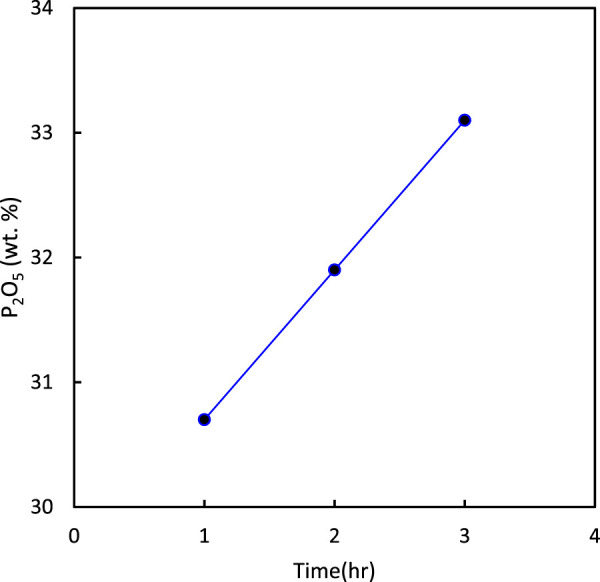
Effect of calcination time on the percentage of P_2_O_5_ in the ore (at 900°C).

**FIGURE 7:**
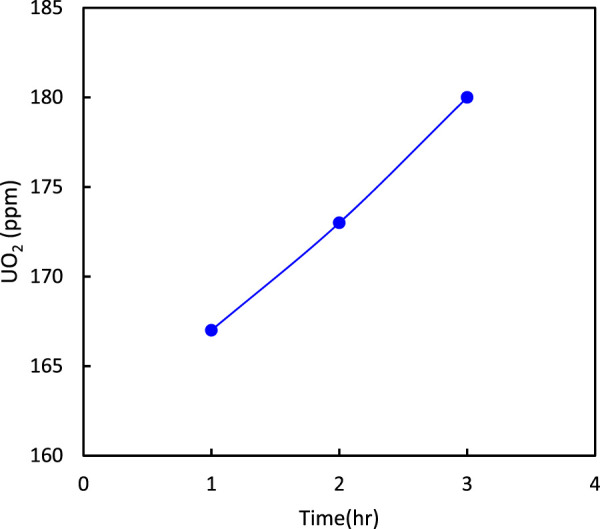
Effect of calcination time on the percentage of UO_2_ in the ore (at 900°C).

The effect of calcination time on P_2_O_5_ and UO_2_ are shown in Figures 6, 7, respectively. The results indicated that an increase in calcination time increased the percentage of P_2_O_5_ and UO_2_ in the ore. From energy consumption and environmental standpoints, a time of 2 h was considered economical.

### 3.3 Flotation

The effect of pH (range 6–10) on the recovery of P_2_O_5_ and UO_2_ in the flotation process was evaluated, see Table 3. The CaO/P_2_O_5_ ratio in the tailing is a very important parameter in the flotation process. The lowest value of this parameter was found at pH = 10. The highest value of UO_2_, P_2_O_5_, and P_2_O_5_ recovery in the tailing part were detected at pH = 10, which is a sign of reverse flotation process. The terms “concentrate” and “tail” were used for the sink fraction and the froth phase, respectively.

**TABLE 3:** Effect of pH on the recovery of P_2_O_5_ and UO_2_ in the flotation process (sodium oleate concentrations = 2,000 g. ton^−1^ of rock).

pH	Weight (%)		P_2_O_5_ (%)		UO_2_ (ppm)		CaO (%)		SiO_2_ (%)		CaO/P_2_O_5_		P_2_O_5_ recovery (%)	
	Tail	Concentrate	Tail	Concentrate	Tail	Concentrate	Tail	Concentrate	Tail	Concentrate	Tail	Concentrate	Tail	Concentrate
6	35	65	27.05	34.27	100	120	57.79	44.97	9.89	7.8	2.13	1.31	32	75.5
8	33.6	66.4	26.2	35.16	100	150	53.46	49.47	9.31	7.45	2.04	1.4	29.8	79.2
10	31	69	25.1	36.1	100	180	55.55	44.64	8.48	7.22	2.21	1.23	26.8	84.4

The effect of sodium oleate concentrations (range 500–2,500 g. ton^−1^ of rock), as a selective collector, on the recovery of P_2_O_5_ and UO_2_ in the flotation process was investigated, see Table 4. By increasing the collector concentration to 2,000 g/ton, the concentration of P_2_O_5_ and the efficiency of P_2_O_5_ recovery in the concentrated part increased, while the CaO/P_2_O_5_ ratio decreased, which indicated reverse flotation. For a collector concentration of 2,500 g/ton, the P_2_O_5_ concentration and its recovery efficiency in the concentrated part decreased, while the CaO/P_2_O_5_ ratio increased. The concentration of SiO_2_ in the concentrated phase was a little less than in the tail part.

**TABLE 4:** Effect of collector (sodium oleate) concentrations on the recovery of P_2_O_5_ and UO_2_ in the flotation process (pH = 10).

Collector concentration (g. ton^−1^ of rock)	Weight (%)		P_2_O_5_ (%)		UO_2_ (ppm)		CaO (%)		SiO_2_ (%)		CaO/P_2_O_5_		P_2_O_5_ recovery (%)	
	Tail	Concentrate	Tail	Concentrate	Tail	Concentrate	Tail	Concentrate	Tail	Concentrate	Tail	Concentrate	Tail	Concentrate
500	23	77	29.1	31.05	130	150	51.98	50.11	8.39	7.14	1.78	1.61	22.6	81
1000	24	76	28.65	32.18	130	150	51.29	48.04	8.4	7.14	1.79	1.49	23.3	82.9
1500	27.2	72.8	27.25	34.05	120	150	52.75	46.24	8.45	6.77	1.93	1.35	25.1	84
2000	31	69	25.1	36.1	100	180	55.55	44.64	8.48	7.22	2.21	1.23	26.8	84.4
2500	31.6	68.4	25.7	35.5	100	200	53.95	45.72	8.51	7.1	2.09	1.28	27.5	83.8

Depressants play a vital role in the uranium flotation process by inhibiting the flotation of undesired materials, thereby improving the efficiency and effectiveness of uranium extraction. These substances form a protective layer on the surface of non-desirable particles, preventing their flotation and allowing for a higher concentration of uranium during the separation process. The appropriate selection and utilization of depressants contribute to cost reduction and lead to improved separation quality in uranium flotation. Overall, depressants are essential for optimizing the performance and productivity of uranium extraction operations. Sodium sulfate, phosphoric acid, citric acid, starch, and sodium silicate were examined as depressants in the flotation process, see Table 5. The results indicated that the weight of concentrated parts was higher than the tail parts. The recovery efficiency of P_2_O_5_ in the concentrated parts was higher than in the tail parts, indicative of the reverse flotation process. The CaO/P_2_O_5_ ratio of the concentrated parts was higher than 1.45. The P_2_O_5_ content in the concentrated parts was under acceptable values. The CaO content in the tail parts was acceptable. For some depressants (such as phosphoric acid), the CaO/P_2_O_5_ ratio was over acceptable values. The best results, shown in Table 5, were obtained using phosphoric acid at pH 5 and the highest P_2_O_5_ recovery with the second highest P_2_O_5_ grade in concentrated parts. However, P_2_O_5_ and UO_2_ were not achieved in significantly high contents in these tests.

**TABLE 5:** Effect of depressant on the recovery of P_2_O_5_ and UO_2_ in the flotation process (collector concentrations = 2,000 g. ton^−1^ of rock).

Depressant	pH	Weight (%)		P_2_O_5_ (%)		UO_2_ (ppm)		CaO (%)		SiO_2_ (%)		CaO/P_2_O_5_		P_2_O_5_ recovery (%)	
		Tail	Concentrate	Tail	Concentrate	Tail	Concentrate	Tail	Concentrate	Tail	Concentrate	Tail	Concentrate	Tail	Concentrate
Sodium sulfate	6	40	460	32.59	32.32	100	150	45.4	50.17	10.47	8.61	1.39	1.55	8	92
	8	130	370	31.81	32.34	100	150	49.04	51.58	9.82	8.21	1.54	1.59	25	75
	10	165	335	31.95	33.63	120	100	50.98	49.99	8.79	8.04	1.59	1.48	33	67
Phosphoric acid	5	32.5	467.5	33.7	33.06	150	100	44.38	48.85	10.29	8.35	1.31	1.48	7	93
Citric acid	8	42.5	457.5	32.24	31.84	150	160	46.39	49.85	10.37	8.52	1.43	1.56	8.5	91.5
starch	8	55	445	31.83	32.94	150	120	48.4	49.78	10.51	8.38	1.52	1.51	10.8	89.2
Sodium silicate	8	65	435	31.73	32.16	100	160	48.36	50.1	11.97	8.61	1.52	1.55	12.7	87.3

The effect of flotation time (2–10 min) on the recovery of P_2_O_5_ and UO_2_ was evaluated, see Table 6. By increasing the flotation time, the recovery of P_2_O_5_ in the tail parts increased and decreased in the concentrated parts. The highest P_2_O_5_ content and the lowest CaO/P_2_O_5_ ratio in the concentrated parts were obtained for 5 min flotation. The UO_2_ content in the tail and concentrated parts was almost constant for all flotation times.

**TABLE 6:** Effect of flotation time on the recovery of P_2_O_5_ and UO_2_ (collector concentrations = 2,000 g. ton^−1^ of rock and pH = 10).

Time	Weight (g)		P_2_O_5_ (%)		UO_2_ (ppm)		CaO (%)		SiO_2_(%)		CaO/P_2_O_5_		P_2_O_5_ recovery (%)	
	Tail	Concentrate	Tail	Concentrate	Tail	Concentrate	Tail	Concentrate	Tail	Concentrate	Tail	Concentrate	Tail	Concentrate
2	121	379	24.1	34.5	100	150	55.10	45.05	8.14	7.35	2.28	1.30	19.7	88.6
5	155	345	25.1	36.1	100	180	55.55	44.64	8.48	7.22	2.21	1.23	26.8	84.4
8	194	306	27.1	35.8	100	180	54.20	45.35	8.5	7.14	2.00	1.26	35.6	74.2
10	217	283	29.5	34.5	120	150	53.20	46.80	8.61	7.04	1.8	1.35	43.4	66.2

### 3.4 Leaching

The effect of the liquid to solid ratio (L/S), sulfuric acid concentration, time, and temperature on the leaching process were evaluated. The optimization of these parameters was performed using the response surface method (RSM) with Design Expert 13 software, employing a central composite design (CCD) approach.

The RSM usually consists of three steps: 1) design and experiments, 2) modeling the response surface through regression, and 3) optimization. The independent parameters and the response are related as follows:

$$y=f\left(x_{1},x_{2},x_{3},\ldots ,x_{n}\right)\;\pm e$$ (4)

where, f is the answer function, y is the answer, e is the test error, and X_n_ is an independent variable. The CCD layout for the optimization of the leaching independent variables is tabulated in Table 7. An empirical relationship between the independent variables and the efficiency of leaching was determined using the RSM, given as:

$$\begin{array}{ll} Y & =-26.66475+\left(1.833\times A\right)+\left(23.833\times B\right)+\left(0.31225\times C\right)-\left(0.546887\times D\right)\\ & -\left(3\times A\times B\right)+\left(0.25\times A\times C\right)+\left(0.1875\times A\times D\right)-\left(0.75\times B\times C\right)+\left(0.025\times B\times D\right)\\ & +\left(0.0375\times C\times D\right)+\left(0.739625\times A^{2}\right)-\left(1.38538\times B^{2}\right)+\left(0.239625\times C^{2}\right)-\left(0.000026\times D^{2}\right) \end{array}$$ (5)

where A is L/S, B is sulfuric acid concentration, C is leaching time, and D is temperature. The statistical results of the RSM are given in Table 8. The predicted and adjusted *R* ^*2*^ values were almost one. The results of the quadratic model in the form of analysis of variance (ANOVA) are given in Table 9. The *p*-value of the model was less than 0.0001, indicating that the model is statistically acceptable.

**TABLE 7:** CCD layout for optimization of leaching independent variables.

Run	L/S (mL/g)	Concentration (molar)	Time (h)	T (^0^C)	Experimental efficiency (%)	Model efficiency (%)	Error (%)
**1**	4	4	3	20	22	23	4.3
**2**	3	5	4	80	42	41.18	1.9
**4**	3	3	2	40	24	22.68	5.5
**5**	5	5	4	40	33	32.18	2.48
**6**	4	4	5	60	44	44.56	1.27
**8**	4	2	3	60	33	34.06	3.21
**10**	5	5	2	40	32	30.68	4.12
**11**	4	4	3	60	39.3	39.75	1.14
**12**	2	4	3	60	26	27.06	4.07
**13**	3	5	2	80	39	37.68	3.38
**14**	3	3	4	40	27	26.18	3.03
**15**	4	4	3	60	39	39.75	1.90
**16**	4	4	3	100	55	56.06	1.92
**17**	5	3	2	80	59	57.68	2.37
**18**	6	4	3	60	57	58.06	1.82
**19**	5	3	4	80	66	65.18	1.24
**20**	4	4	1	60	35	36.56	4.20
**21**	4	6	3	60	33	34.06	3.11

**TABLE 8:** Statistical results of fit summary in CCD.

Source	Sequential *p*-value	Lack of fit *p*-value	Adjusted *R* ^2^	Predicted *R* ^2^	Comments
Linear	<0.0001	0.0004	0.9076	0.8507	Suggested
2FI	0.2155	0.0005	0.9271	0.6910	—
Quadratic	0.0270	0.0019	0.9758	0.1350	Suggested
Cubic	0.0019	—	0.9984	—	Aliased

**TABLE 9:** Analysis of variance (ANOVA) for the quadratic model.

Source	Sum of squares	df	Mean square	F-value	*p*-value	Comments
Model	2724.04	14	194.57	58.54	<0.0001	Significant
Residual	19.94	6	3.32	—	—	—
Lack of fit	19.06	2	9.53	43.31	0.0019	Significant
Pure error	0.88	4	0.22	—	—	—
Cor total	2743.98	20	—	—	—	—

#### 3.4.1 Effect of L/S ratio and sulfuric acid concentration

**FIGURE 8:**
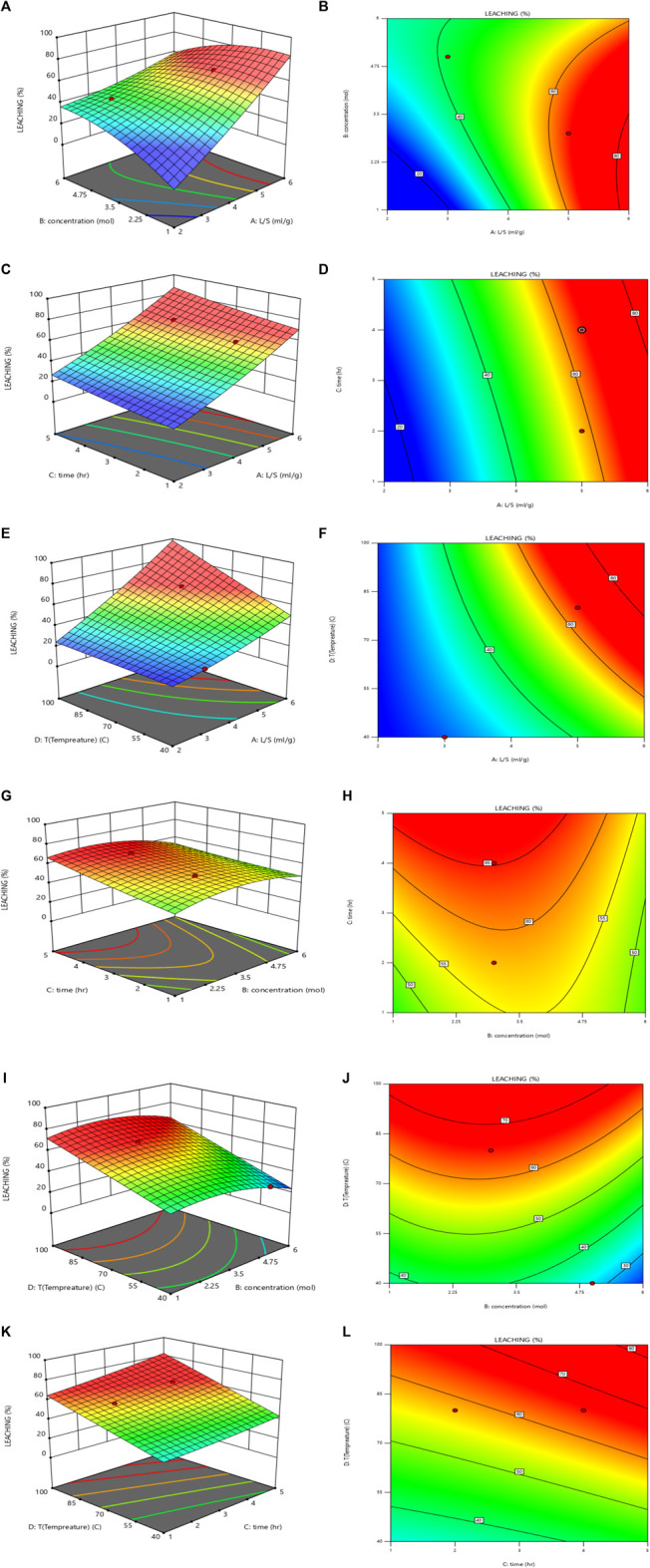
Effect of L/S ratio and sulfuric acid concentration on the leaching efficiency (T = 80°C, time = 4 h): **(A)** 3D plot and **(B)** contour plot, Effect of the L/S ratio and time on the leaching efficiency (T = 80°C, sulfuric acid concentration = 3 M): **(C)** 3D plot and **(D)** contour plot, Effect of L/S ratio and temperature on the leaching efficiency (time = 4 h, sulfuric acid concentration = 3 M): **(E)** 3D plot and **(F)** contour plot, Effect of sulfuric acid concentration and time on the leaching efficiency (T = 80°C, L/S = 5): **(G)** 3D plot and **(H)** contour plot, Effect of sulfuric acid concentration and temperature on the leaching efficiency (time = 4 h, L/S = 5): **(I)** 3D plot and **(J)** contour plot and Effect of time and temperature on the leaching efficiency (sulfuric acid concentration = 3 M, L/S = 5): **(K)** 3D plot and **(L)** contour plot.

The RSM was used to investigate the simultaneous effect of the L/S ratio (2–6 mL/g) and sulfuric acid concentration (1–6 M) on the leaching efficiency. As shown in Figures 8A, B, for 3–4 M sulfuric acid concentration, with an increase in the L/S ratio, the uranium extraction efficiency increased.

#### 3.4.2 Effect of L/S ratio and time

The simultaneous effect of the L/S ratio (2–6 mL/g) and time (1–6 h) on the leaching efficiency was examined using the RSM. As shown in Figures 8C, D, with a simultaneous increase in the L/S ratio and time, the uranium leaching efficiency increased. The result indicated that the optimum values of the L/S ratio and time are 5 mg/L and 4 h, respectively.

#### 3.4.3 Effect of L/S ratio and temperature

The simultaneous effect of the L/S ratio (2–6 mL/g) and temperature (20°C–100°C) on the leaching efficiency is shown in Figures 8E, F. The uranium leaching efficiency increased with the simultaneous increase in the L/S ratio and temperature. 80°C was determined as the optimum temperature.

#### 3.4.4 Effect of acid concentration and time

The simultaneous effect of sulfuric acid concentration (1–6 M) and time (1–6 h) on the leaching efficiency is shown in Figures 8G, H. For 1–4 M sulfuric acid concentration, the uranium leaching efficiency increased with an increase in the sulfuric acid concentration. For 4–6 M sulfuric acid concentration, by increasing the sulfuric acid concentration, uranium was co-precipitated with gypsum, and the uranium leaching efficiency decreased. The uranium leaching efficiency increased with an increase in time, and 4 h was determined to be the optimum leaching time.

#### 3.4.5 Effect of acid concentration and temperature

The simultaneous effect of sulfuric acid concentration (1–6 M) and temperature (20°C–100°C) on the leaching efficiency is shown in Figures 8I, J. For 3–4 M sulfuric acid concentration, with an increase in temperature, the uranium extraction efficiency increased. As a confirmation of the results presented in Section 3.4.3, 80°C was determined to be the optimum temperature.

#### 3.4.6 Effect of time and temperature

The simultaneous effect of time (1–6 h) and temperature (20°C–100°C) on the leaching efficiency is shown in Figures 8K, L). The uranium leaching efficiency increased with a simultaneous increase in the time and temperature.

#### 3.4.7 Model confirmation

To confirm the presented RSM model, leaching experiments were performed. The uranium leaching efficiency predicted by the RSM model, experimental efficiency, and error rate are given in Table 10. The errors of the model response were less than 10%, which indicate the accuracy of the presented RSM model.

**TABLE 10:** Uranium leaching efficiency predicted by RSM model in comparison with the experimental efficiency.

Leaching parameters	Response of experimental (%)	Response of model (%)	Error (%)
Time (h) = 4	43.9	48.58	9.6
Concentration (M) = 4			
L/S (mL/g) = 4			
Temperature (°C) = 75			
Time (h) = 4	27.33	29.32	6.7
Concentration (M) = 4			
L/S (mL/g) = 3			
Temperature (°C) = 40			
Time (h) = 4	31	28.78	7.1
Concentration (M) = 2			
L/S (mL/g) = 4			
Temperature (°C) = 80			
Time (h) = 5	49.8	54.3	7.7
Concentration (M) = 4			
L/S (mL/g) = 4			
Temperature (°C) = 80			
Time (h) = 4	66	63.3	4
Concentration (M) = 4			
L/S (mL/g) = 5			
Temperature (°C) = 80			
Time (h) = 4	37.7	34.58	8.2
Concentration (M) = 4			
L/S (mL/g) = 3			
Temperature (°C) = 60			

According to the RSM modeling and experimental results, the optimum parameters of uranium leaching from Sheikh Habil-Iran phosphate ore are presented in Table 11. The experimental results confirmed the optimum leaching parameters determined by the RSM model.

**TABLE 11:** Optimum value of leaching parameters.

Parameters	L/S	Time(h)	Concentration (M)	Temperature (°C)
Suggested by RSM	5	4	3	80
Experimental result	5	4	3	80

#### 3.4.8 Effect of pre-treatment methods on uranium leaching efficiency

**FIGURE 9:**
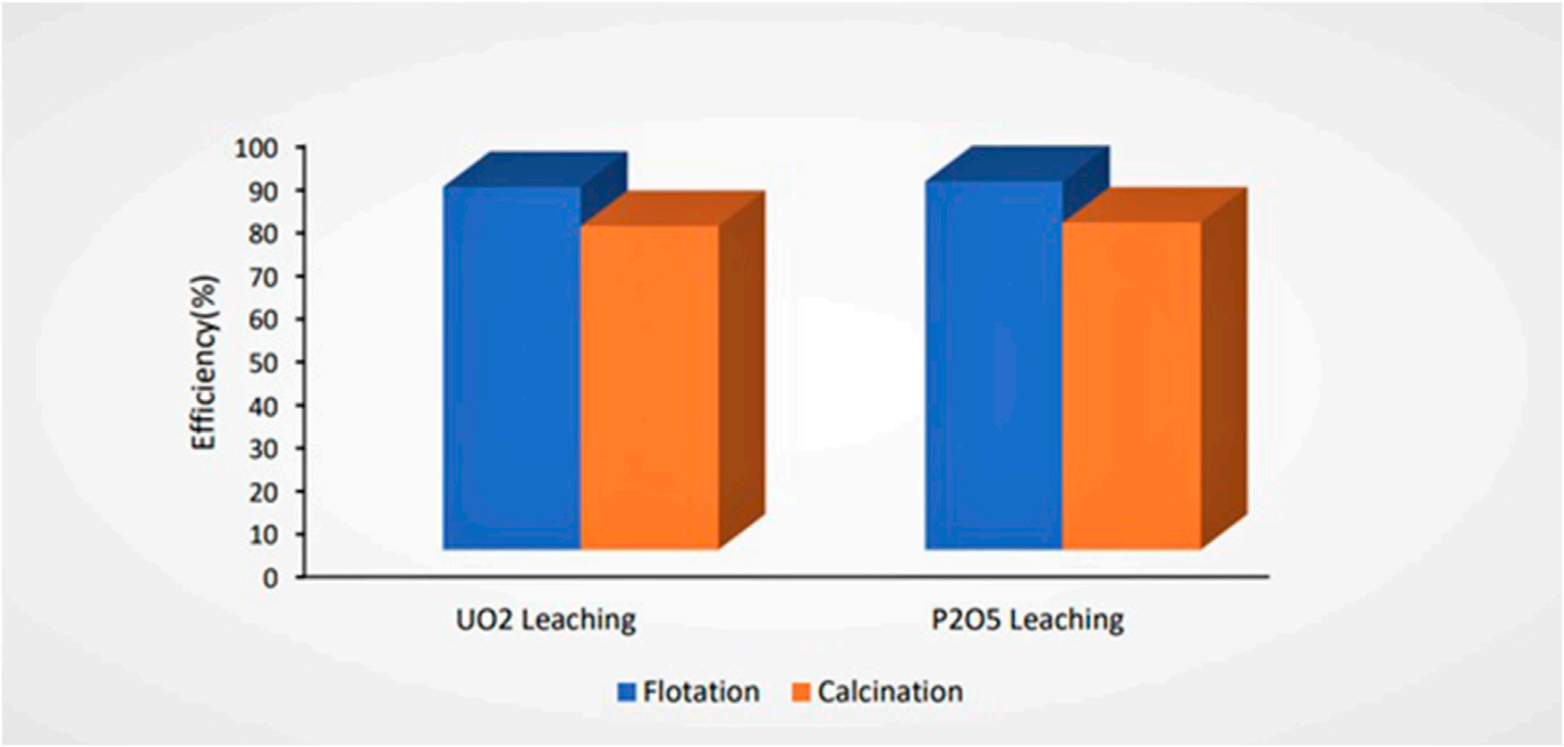
Effect of pre-treatment methods on uranium leaching efficiency (L/S = 5, time = 4 h, sulfuric acid concentration = 3 M, and T = 80°C).

The UO_2_ and P_2_O_5_ extraction efficiencies from pre-treated ores by flotation and calcination leached under optimum leaching parameters are shown in Figure 9. The uranium extraction efficiency from ore pre-treated by flotation was higher than that for ore pre-treated by calcination.

### 3.5 Phosphoric acid production

**FIGURE 10:**
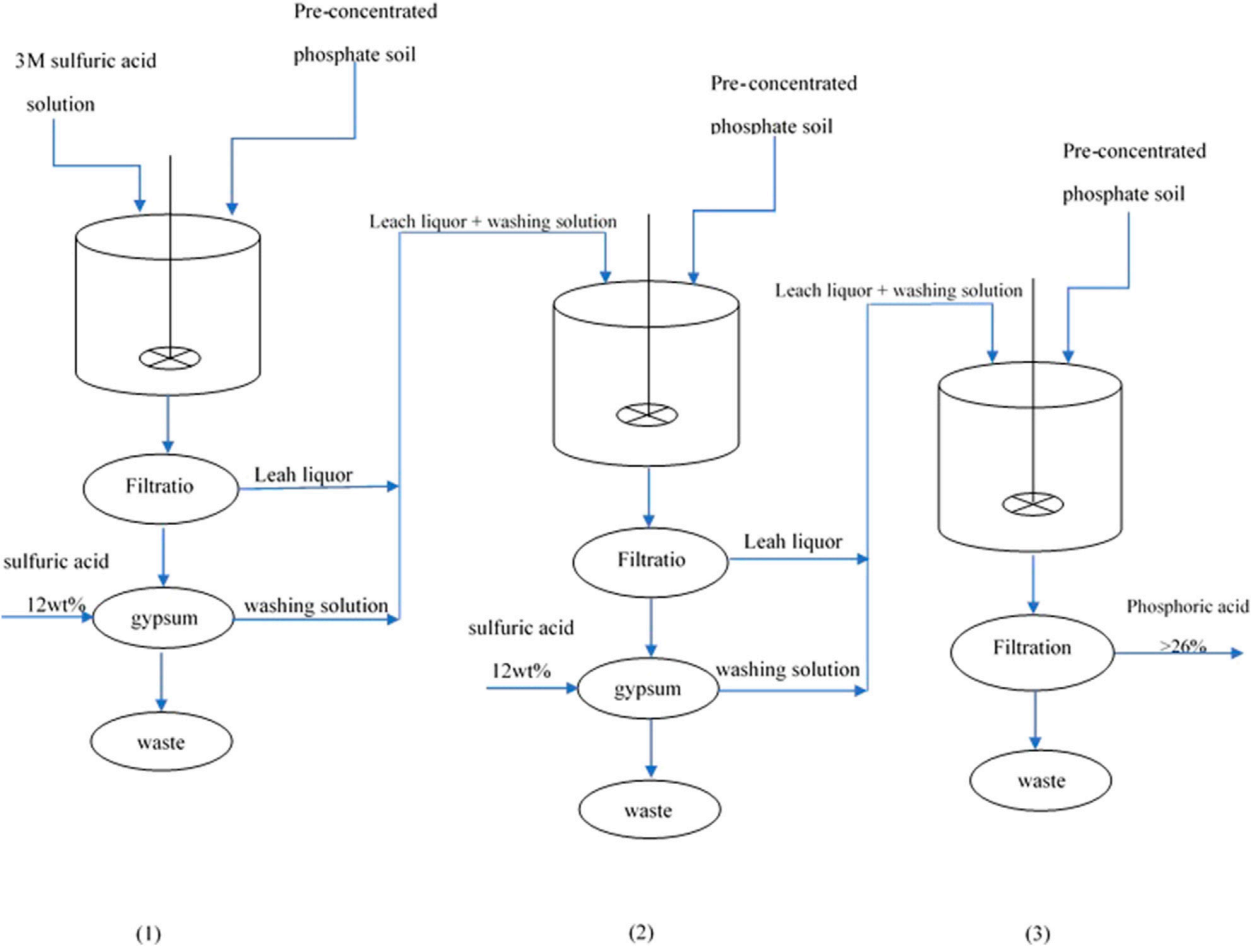
Schematic of the three sequential leaching steps for the production of phosphoric acid with a grade of 26 wt% P_2_O_5_.

In order to produce phosphoric acid with a concentration exceeding 55%, which is achieved through the evaporation process, it is necessary to produce intermediate phosphoric acid with a concentration greater than 26%. Such a process consisting of three sequential leaching steps, under optimum parameters determined by the RSM model, was designed, see Figure 10. At the end of steps 1 and 2, the remaining gypsum was washed with 12 wt% sulfuric acid. The leach liquor and washing solution of these two steps was fed into the next step. The characteristics of the three leaching steps are given in Table 12. The UO_2_ and H_3_PO_4_ extraction efficiencies of this process were 88.3% and 91.4%, respectively. The uranium and H_3_PO_4_ concentrations of the produced phosphoric acid were 70 ppm and 28.2 wt%, respectively.

**TABLE 12:** Characteristics of the three leaching steps for phosphoric acid production.

Step	U (ppm)	H_3_PO_4_ (%)	UO_2_ efficiency (%)	H_3_PO_4_ efficiency (%)
1	45.7	11.4	84.2	85.5
2	59.3	19.5	85.7	87.1
3	75.6	28.2	88.3	91.4

## 4 Conclusion

In the present study, the concentration process for phosphate ore of the Sheikh Habil-Iran mine was investigated using flotation and calcination methods, and the process parameters were optimized. Next, the pre-treated phosphate ore was leached using sulfuric acid. The leaching parameters were optimized to produce a suitable grade of phosphoric acid and to maximize uranium recovery using the response surface methodological approach (RSM). According to the RSM modeling and experimental results, the optimum parameters for uranium leaching from the Sheikh Habil-Iran phosphate ore were an L/S = 5 mL/g, sulfuric acid concentration = 3 M, time = 4 h, and temperature = 80°C. The optimized efficiencies of uranium recovery from phosphate ore pre-treated by flotation and calcination methods were 84.2% and 75.2%, respectively. The results indicated that flotation has superiority over calcination as a pre-treatment method for phosphate ore of the Sheikh Habil-Iran mine. For the production of phosphoric acid with a grade of 26 wt% P_2_O_5_, a process consisting of three sequential leaching steps was designed. The UO_2_ and H_3_PO_4_ extraction efficiencies of this process were 88.3% and 91.4%, respectively. The uranium and H_3_PO_4_ concentrations of the produced phosphoric acid were 70 ppm and 28.2 wt%, respectively.

## Data Availability

The datasets presented in this study can be found in online repositories. The names of the repository/repositories and accession number(s) can be found in the article/supplementary material.
